# Association between inflammatory bowel disease and cancer risk: evidence triangulation from genetic correlation, Mendelian randomization, and colocalization analyses across East Asian and European populations

**DOI:** 10.1186/s12916-024-03352-9

**Published:** 2024-03-25

**Authors:** Di Liu, Meiling Cao, Haotian Wang, Weijie Cao, Chenguang Zheng, Yun Li, Youxin Wang

**Affiliations:** 1 Centre for Biomedical Information Technology, Shenzhen Institutes of Advanced Technology, Chinese Academy of Sciences, Shenzhen, 518055 China; 2 Beijing Key Laboratory of Clinical Epidemiology, School of Public Health, Capital Medical University, Beijing, 100069 China; 3 Centre for Precision Medicine, Edith Cowan University, Perth, WA7027 Australia; 4 School of Public Health, North China University of Science and Technology, Tangshan, 063210 China; 5 Hebei Key Laboratory of Organ Fibrosis, North China University of Science and Technology, Tangshan, 063210 China

**Keywords:** Inflammatory bowel disease, Cancers, Mendelian randomization, Genetic correlation, Colocalization analysis

## Abstract

**Background:**

Inflammatory bowel disease (IBD), which includes Crohn’s disease (CD) and ulcerative colitis (UC), has been associated with several cancer risks in observational studies, but the observed associations have been inconsistent and may face the bias of confounding and reverse causality. The potential causal relationships between IBD and the risk of cancers remain largely unclear.

**Methods:**

We performed genome-wide linkage disequilibrium score regression (LDSC), standard two-sample Mendelian randomization (MR), and colocalization analyses using summary genome-wide association study (GWAS) data across East Asian and European populations to evaluate the causal relationships between IBD and cancers. Sensitivity analyses for the MR approach were additionally performed to explore the stability of the results.

**Results:**

There were no significant genetic correlations between IBD, CD, or UC and cancers (all *P* values > 0.05) in East Asian or European populations. According to the main MR analysis, no significant causal relationship was observed between IBD and cancers in the East Asian population. There were significant associations between CD and ovarian cancer (odds ratio [OR] = 0.898, 95% CI = 0.844–0.955) and between UC and nonmelanoma skin cancer (OR = 1.002, 95% CI = 1.000–1.004, *P* = 0.019) in the European population. The multivariable MR analysis did not find any of the above significant associations. There was no shared causal variant to prove the associations of IBD, CD, or UC with cancers in East Asian or European populations using colocalization analysis.

**Conclusions:**

We did not provide robust genetic evidence of causal associations between IBD and cancer risk. Exposure to IBD might not independently contribute to the risk of cancers, and the increased risk of cancers observed in observational studies might be attributed to factors accompanying the diagnosis of IBD.

**Supplementary Information:**

The online version contains supplementary material available at 10.1186/s12916-024-03352-9.

## Background

Cancers, which are important causes of morbidity and mortality and carry an enormous disease burden, remain a formidable challenge [1]. An increasing number of studies have shown that the incidence of cancer in various organs diagnosed in adults younger than 50 years old has been increasing globally [2–4]. Therefore, identifying risk factors in young people is crucial for preventing early-stage cancer.

Inflammatory bowel disease (IBD), which includes Crohn’s disease (CD) and ulcerative colitis (UC) and is more common in young people, is a debilitating and progressive disorder of the gastrointestinal tract [5]. The symptoms of IBD patients, such as abdominal pain, diarrhea, bloody stool, and frequent bowel movements, seriously affect quality of life, affecting millions of people worldwide and increasing in prevalence [5–7]. IBD is a chronic disorder characterized by intestinal inflammation that can induce adverse outcomes [8].

Because IBD is difficult to treat, there is an increased risk of cancer without formal treatment [9]. The well-established connection between IBD and intestinal cancer, particularly colorectal cancer (CRC), is supported by numerous observational studies [10–12]. This heightened risk of intestinal cancer may be attributed to long-term chronic inflammation [13]. Observational studies have also identified associations between IBD and extraintestinal cancer, such as skin, hepatobiliary, and lung cancer [12, 14]. However, traditional observational studies have found it challenging to mitigate the biases caused by confounding and reverse causation. In addition, an umbrella review and reanalysis of meta-analyses of observational studies have indicated that associations between IBD and different cancers are inconsistent [15]. Despite several studies that have examined the potential causal associations between IBD and cancer risk by using the Mendelian randomization (MR) approach [16–20], which can overcome the limitations of traditional observational study designs by using Mendel’s law, most of the MR studies were based on European population, and only one MR study explored the association between IBD and hepatobiliary pancreatic cancer in the East Asian population [18]. However, whether lifelong exposure to IBD has a causal association with cancers is still largely unknown.

Summarized data derived from genome-wide association studies (GWAS) are widely reported to make two-sample MR studies easy and popular to implement [21, 22]. A recent GWAS with a larger sample size reported loci associated with IBD in East Asian populations [23], which allowed us to explore the associations between IBD and cancers in the East Asian population. Although the MR approach utilizes the random allocation of genotypes during meiosis to yield natural randomized controlled trials, effectively mitigating potential unmeasured confounding and reverse causation [24, 25], this approach still faces some challenges.

Recently, increasing attention has been given to combining genetic correlation with MR analyses [26, 27] or combining MR with colocalization analyses [28, 29] as analytical frameworks to illuminate causal associations. Linkage disequilibrium score regression (LDSC) enables the assessment of genetic correlation between two traits, offering a broad range of genetic insights without being affected by sample overlap; its principal limitation lies in its incapacity to infer causality [30]. MR analysis provides evidence similar to randomized controlled trial [24], but the drawback is the need to satisfy certain assumptions and susceptibility to pleiotropy and weak instrumental variable bias. Colocalization analysis can ascertain whether two or more traits or diseases share the same genetic variants as their potential causal variants, offering specific genetic variant information and assessing potential pleiotropy; however, this approach is limited by its statistical power [31].

Therefore, we integrated genetic correlation, MR, and colocalization approaches to investigate the causal relationship between IBD and cancer risk. This is the first study using such a comprehensive approach, balancing the genetic evidence from these three methods to provide a robust understanding of the causal association between IBD and cancer risk across East Asian and European populations.

## Methods

### Study design

**Fig. 1:**
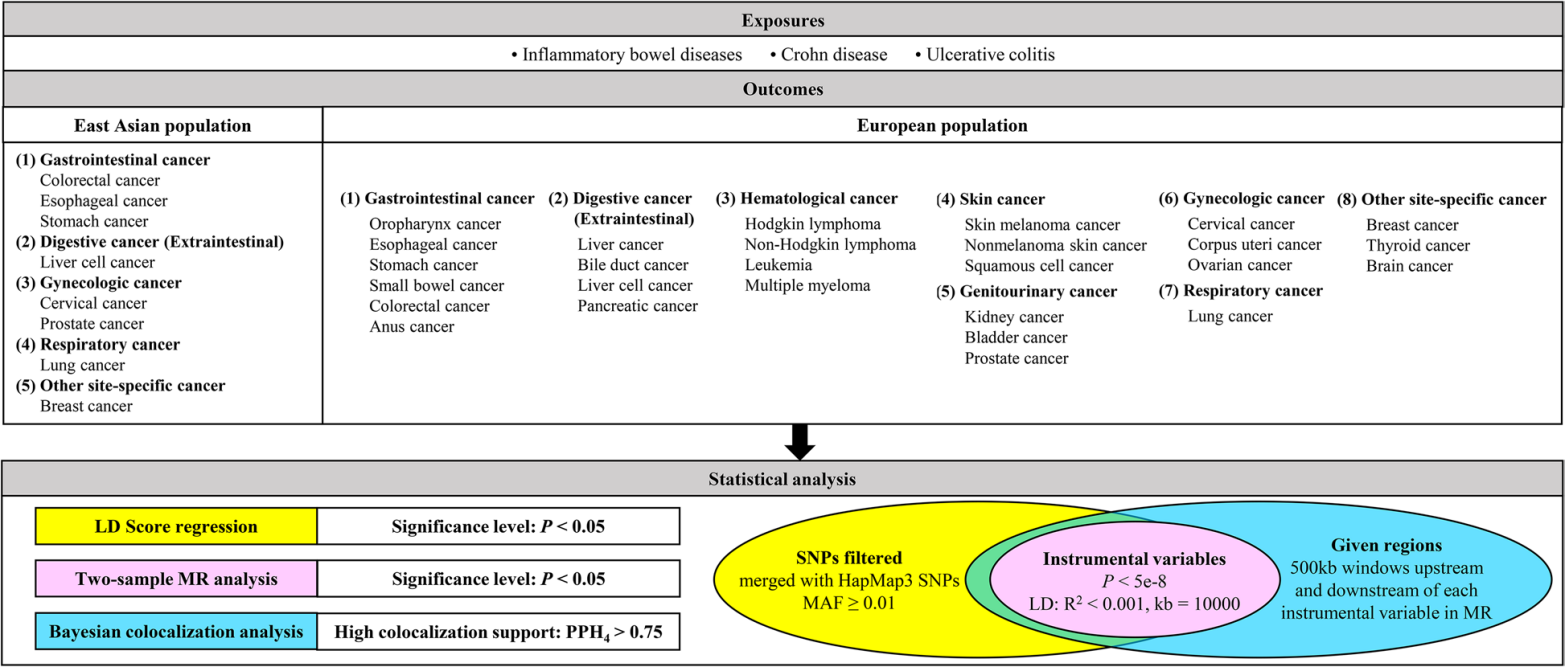
An overview of the study design and process. LD, linkage disequilibrium; MAF, minor allele frequency; MR, Mendelian randomization

Our study was based on summary-level GWAS data available for East Asian and European populations to explore the potential causal associations between IBD (including UC and CD) and the risk of cancers. In the East Asian population, eight site-specific cancers were included in the analysis: colorectal, esophageal, stomach, liver cell, cervical, prostate, lung, and breast cancers. In the European population, 27 site-specific cancers were selected, including oropharynx, esophageal, stomach, small bowel, colorectal, anus, liver, bile duct, liver cell, pancreatic, Hodgkin lymphoma, non-Hodgkin lymphoma, leukemia, multiple myeloma, skin melanoma, nonmelanoma skin, squamous cell, kidney, bladder, prostate, cervical, corpus uteri, ovarian, lung, breast, thyroid, and brain cancers. Genome-wide LDSC was used to assess the genetic association between IBD and cancer. A standard two-sample MR analysis was performed to clarify the causal relationship between IBD and cancer. Colocalization analysis was used to investigate the local genetic structure shared between IBD and cancer and to assess whether the causal association was due to chance. Figure 1 provides an overview of the design and process of our analysis.

Our study followed the STROBE-MR guidelines [22]. The STROBE-MR checklist is available in Additional file 1: Table S1.

### Data sources

#### GWAS data for IBD

The GWAS summary-level data for IBD/UC/CD patients based on East Asian and European populations were released in the published study [23, 32]. The meta-GWAS for the East Asian population included 14,393 patients with IBD and 15,456 controls, 7372 patients with CD and 14,946 controls, and 6862 patients with UC and 15,456 controls [23]. The meta-GWAS for the European population included 25,042 patients with IBD and 34,915 healthy controls, 12,194 with CD and 28,072 healthy controls, and 12,366 with UC and 33,609 healthy controls [32]. More detailed information can be found in Additional file 1: Table S2.

#### GWAS data for cancers

GWAS summary statistics of eight cancers for the East Asian population were obtained from a large-scale GWAS conducted by Kazuyoshi Ishigaki et al. [33] from the Biobank Japan. The samples were collected from 12 medical institutions across Japan and included approximately 200,000 participants. The sample size of the GWAS data for cancers ranged from 90,336 to 212,453, and the number of cases ranged from 605 to 7062. More details of the GWAS data for cancers are listed in Additional file 1: Table S2.

With respect to the European population, we used data from the latest available GWAS data, which had the largest sample size or the largest sample size of patients for the outcome under investigation. The GWAS summary statistics for 27 cancers were obtained mainly from the following sources: (i) Rashkin SR et al. conducted GWAS across 18 types of cancer within two population-based cohorts: the UK Biobank and the Kaiser Permanente Genetic Epidemiology Research on Adult Health and Aging cohorts [34]; (ii) Jiang L et al. utilized fastGWA-GLMM to the UK Biobank data and subsequently procured full summary statistics [35]; (iii) A study conducted by Kimberley Burrows et al. presented detailed information on the GWAS focusing on pan-cancer and site-specific cancers among participants from the UK Biobank [36]; (iv) Meta-analysis with Transdisciplinary Research of Cancer in Lung of the International Lung Cancer Consortium and Lung Cancer Cohort Consortium, performed by McKay JD et al. [37]; and (v) Seviiri M et al. executed a multitrait genetic analysis of more than 300,000 participants from Europe, Australia, and the USA [38]. The sample size of the GWAS data ranged from 85,716 to 456,348, and the number of cases ranged from 104 to 29,266. More details of the GWAS data for cancers are listed in Additional file 1: Table S2.

### Data analysis

#### Linkage disequilibrium score regression

Genome-wide LDSC [30] was used to assess the genetic association between IBD and cancer (https://github.com/bulik/ldsc). The LDSC calculates genetic correlation by considering the impact of all single nucleotide polymorphisms (SNPs), even those that do not achieve genome-wide significance. We removed SNPs that did not merge with HapMap3 SNPs and those with a minor allele frequency less than 0.01. The findings are presented as genetic correlation (*r*_*g*_) with standard error (SE). The results of LDSC analysis could not be available if either one or both traits exhibited too low heritability [39, 40].

*P* values less than 0.05 were considered suggestive of evidence for a potential genetic correlation. Statistical analysis was performed using LDSC v1.0.1.

#### Mendelian randomization analysis

MR analysis is an instrumental variable analysis that uses genetic variants as instrumental variables (IVs) to study causality. Additional file 2: Fig. S1 provides an overview of our MR design. MR analysis is based on three main assumptions: (i) genetic instruments are associated with exposure, (ii) genetic instruments are independent of any confounder, and (iii) genetic instruments affect outcome only through exposure.

Conditionally uncorrelated variants strongly (*P* < 5 × 10^−8^) and independently (linkage disequilibrium [LD] *r*^2^ < 0.001, window size = 10,000 kb) associated with IBD/UC/CD were extracted as IVs. The LD proxies were defined using 1000 genomes from East Asian and European samples. We calculated the overall *R*^2^ and *F*-statistics by summing the estimated *R*^2^ [*R*^2^ = 2 × EAF × (1-EAF) × beta^2^] and *F*-statistics [*F* = beta^2^/se^2^] for each SNP. The *F*-statistics for all traits under consideration exceeded 10 [41], indicating no potential weak instrument bias. In addition, the mRnd website tool (https://shiny.cnsgenomics.com/mRnd/) was used to calculate the statistical power of the MR analysis.

We selected the random-effects inverse-variance weighted (IVW) method as the primary analysis, and sensitivity analyses, including the weighted median (WM), penalized weighted median (PWM), MR-Egger, MR pleiotropy residual sum and outlier (PRESSO), and MR-robust adjusted profile score (RAPS) analyses, were performed to further explore the stability of the results. In addition, the intercepts of the MR-Egger analysis and MR-PRESSO global test were calculated to evaluate pleiotropy. When the global test *P* values in the MR-PRESSO analysis were less than 0.05, the MR-PRESSO estimates were the results after outlier removal. We conducted a Steiger directionality test to rule out potential reverse causality.

In addition, to further avoid potential pleiotropy, we scanned PhenoScanner (on February 6th, 2024; http://www.phenoscanner.medschl.cam.ac.uk) for identifying traits associated with instrumental variables (*R*^2^ ≥ 0.8, *P* values ≤ 5 × 10^−8^), and performed MR after removing SNPs associated with confounding factors (body mass index, waist circumference, hip circumference, waist-hip ratio, percentage of body fat, smoking, alcohol consumption, insomnia, depression, and physical activity). For significant MR findings, we also conducted multivariable MR (MVMR) analysis to obtain estimates independent of these confounding factors.

Pleiotropy poses a challenge to interpreting MR results. Therefore, we reported the primary IVW results, combined with methods for detecting and correcting for pleiotropy, to fully account for the bias from pleiotropy.

*P* values less than 0.05 were considered suggestive of evidence for a potential causal association. The IVW method, sensitivity analyses (excluding MR-RAPS), Steiger directionality test, and MVMR were implemented using the “TwoSampleMR” (version 0.5.7) package in R version 4.3.1. The MR-RAPS analysis was performed using the “mr.raps” (version 0.2) package in R version 4.3.1.

#### Bayesian colocalization analysis

We used this method to assess whether two associated traits were consistent with shared causal variant(s) according to the included IVs. Five mutually exclusive hypotheses were tested: (1) there is no causal genetic variant for either trait (H_0_); (2) there is one causal genetic variant for trait 1 only (H_1_); (3) there is one causal genetic variant for trait 2 only (H_2_); (4) there are two distinct causal genetic variants, one for each trait (H_3_); and (5) there is a causal genetic variant for both traits (H_4_). The posterior probability (PP) is used to quantify the support of each hypothesis and is expressed as PPH_0_, PPH_1_, PPH_2_, PPH_3_, and PPH_4_ [31]. We selected regions that had 500 kb windows upstream and downstream of each instrumental variable in MR for analysis, and the average value of PPH_4_ across all regions was taken as the final colocalization result.

A PPH_4_ level greater than 75% was considered suggestive of evidence for a causal genetic variant for both traits. These PPs were calculated using the “coloc (version 5.2.3)” package in R version 4.3.1.

### Possible results and explanations

**Fig. 2:**
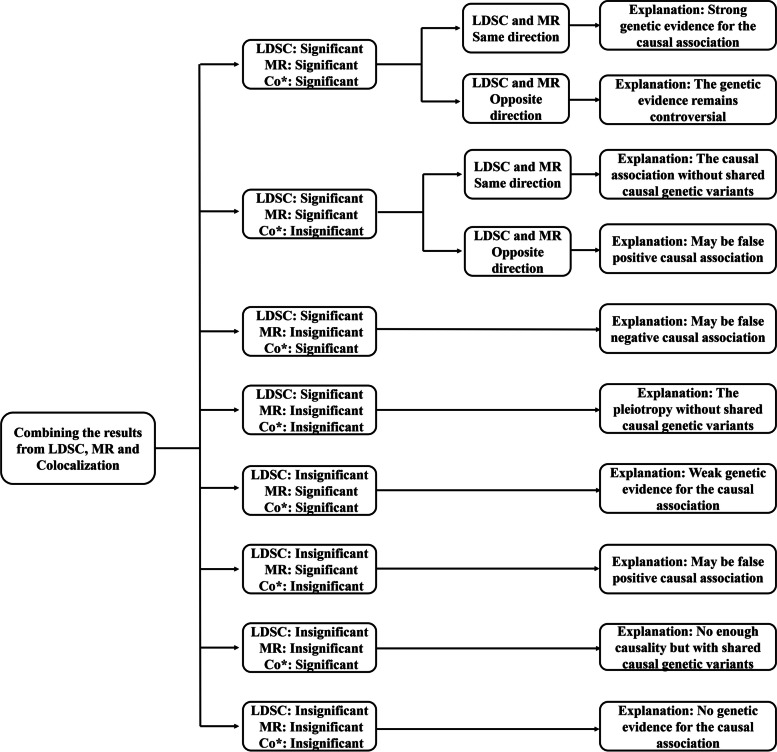
Summarize possible results and explanations. *P* values less than 0.05 in the LDSC and MR analyses were considered suggestive of evidence for a potential association, and a PPH_4_ level greater than 75% was considered suggestive of evidence for a causal genetic variant for both traits. Co*, colocalization; LDSC, linkage disequilibrium score regression; MR, Mendelian randomization

As shown in Fig. 2, we summarized ten possible results and nine explanations combining the results from genetic correlation, MR, and colocalization analyses based on the effects and levels of statistical significance/direction. The results from genetic correlation and MR analyses performed both statistically significant and direct, while colocalization analysis had only statistically significant. The results of MR comprehensively considered the primary analysis of the IVW method and excluded the potential bias of pleiotropy.

The specific results and explanations used were as follows: (Explanation i) when all three results were significant and in the same direction, it was interpreted as strong genetic evidence for the causal association; (Explanation ii) when all three results were significant and in the opposite direction, it was interpreted that the genetic evidence remains controversial; (Explanation iii) when the results from genetic correlation and MR analyses were significant and in the same direction, it was interpreted as the causal association without shared causal genetic variants; (Explanation iv) when the results from genetic correlation and MR analyses were significant and in the opposite direction or when only the result from MR analysis was significant, it might be a false positive causal association; (Explanation v) when the results from genetic correlation and colocalization analyses were significant, it might be a false negative causal association; (Explanation vi) when only the result from LDSC analysis was significant, it was interpreted as pleiotropy without shared causal genetic variants; (Explanation vii) when the results from MR and colocalization analyses were significant, it was interpreted as weak genetic evidence for causal association; (Explanation viii) when only the result from colocalization analysis was significant, it was interpreted as no enough causality but with shared causal genetic variants; (Explanation ix) when all three results were insignificant, it was interpreted as no genetic evidence for the causal association.

### Risk of bias assessment

To assess the quality of the MR studies, we considered 8 potential biases: (1) weak instrument bias, (2) pleiotropy bias, (3) bias from sample overlap, (4) bias from crowd stratification, (5) bias from inconsistency with sensitivity analyses, (6) bias from lack of repeatability, (7) bias from inconsistency with other study design evidence, and (8) reporting bias. Each domain was judged as having a low, moderate (no information was classified as moderate bias), or high risk of bias. The detailed risk bias assessment criteria used in the Mendelian randomization studies can be found in Additional file 1: Table S3.

## Results

### Genetic correlation between IBD and cancers

As shown in Table 1, the genetic correlations of IBD, UC, and CD with cancer in the East Asian population ranged from 0.009 to 0.202. There were no significant genetic correlations between IBD, CD, or UC and cancer (all *P* values > 0.05).

**Table 1:** The genetic correlations between IBD and cancers caused by LDSC in the East Asian population

Outcomes	IBD			CD			UC		
	***r***_***g***_	**se**	***P***	***r***_***g***_	**se**	***P***	***r***_***g***_	**se**	***P***
Colorectal cancer	− 0.082	0.080	0.305	− 0.151	0.078	0.052	− 0.022	0.099	0.820
Esophageal cancer	− 0.040	0.069	0.565	− 0.099	0.071	0.161	− 0.013	0.084	0.879
Stomach cancer	0.073	0.075	0.334	0.069	0.073	0.340	0.025	0.089	0.778
Liver cell cancer	NA	NA	NA	NA	NA	NA	NA	NA	NA
Cervical cancer	− 0.140	0.112	0.214	− 0.202	0.122	0.096	− 0.069	0.126	0.586
Prostate cancer	− 0.062	0.072	0.388	− 0.084	0.078	0.283	− 0.022	0.087	0.804
Lung cancer	− 0.009	0.120	0.941	− 0.051	0.125	0.683	− 0.025	0.146	0.864
Breast cancer	− 0.049	0.066	0.452	− 0.088	0.068	0.194	− 0.022	0.078	0.776

*CD* Crohn’s disease, *IBD* inflammatory bowel disease, *LDSC* linkage disequilibrium score regression, *NA* not available, *UC* ulcerative colitis

As shown in Table 2, the genetic correlations of IBD, UC, and CD with cancer ranged from 0.0004 to 0.133 in the European population. There were no significant genetic correlations between IBD, CD, or UC and cancer (all *P* values > 0.05).

**Table 2:** The genetic correlations between IBD and cancers caused by LDSC in the European population

Outcomes	IBD			CD			UC		
	***r***_***g***_	**se**	***P***	***r***_***g***_	**se**	***P***	***r***_***g***_	**se**	***P***
Oropharynx cancer	NA	NA	NA	NA	NA	NA	NA	NA	NA
Esophageal cancer	0.057	0.036	0.110	0.042	0.034	0.217	0.054	0.043	0.214
Stomach cancer	NA	NA	NA	NA	NA	NA	NA	NA	NA
Small bowel cancer	− 0.066	0.138	0.636	− 0.092	0.166	0.579	0.022	0.119	0.855
Colorectal cancer	− 0.032	0.045	0.476	− 0.017	0.043	0.685	− 0.030	0.053	0.566
Anus cancer	0.011	0.035	0.748	0.043	0.037	0.244	− 0.003	0.043	0.954
Liver cancer	0.035	0.100	0.727	− 0.008	0.089	0.925	0.133	0.316	0.673
Bile duct cancer	0.011	0.052	0.830	0.042	0.063	0.510	0.026	0.069	0.702
Liver cell cancer	− 0.026	0.031	0.397	− 0.031	0.030	0.310	− 0.029	0.038	0.452
Pancreatic cancer	0.038	0.045	0.395	0.002	0.044	0.974	0.052	0.050	0.299
Hodgkin lymphoma	NA	NA	NA	NA	NA	NA	NA	NA	NA
Non-Hodgkin lymphoma	− 0.026	0.033	0.417	− 0.017	0.033	0.612	− 0.044	0.038	0.250
Leukemia	− 0.056	0.046	0.225	− 0.040	0.043	0.353	− 0.020	0.053	0.714
Multiple myeloma	0.004	0.030	0.898	− 0.024	0.030	0.434	0.037	0.039	0.338
Skin melanoma cancer	0.002	0.020	0.939	0.006	0.019	0.772	0.003	0.026	0.909
Nonmelanoma skin cancer	− 0.001	0.012	0.935	0.006	0.013	0.657	− 0.004	0.016	0.790
Squamous cell cancer	0.005	0.009	0.527	0.0004	0.009	0.961	0.014	0.011	0.204
Kidney cancer	NA	NA	NA	NA	NA	NA	NA	NA	NA
Bladder cancer	0.060	0.036	0.092	0.047	0.035	0.175	0.055	0.040	0.163
Prostate cancer	− 0.024	0.016	0.135	− 0.012	0.015	0.429	− 0.030	0.019	0.126
Cervical cancer	0.002	0.029	0.944	− 0.005	0.028	0.846	0.015	0.032	0.645
Corpus uteri cancer	− 0.064	0.062	0.306	− 0.038	0.056	0.504	− 0.088	0.080	0.270
Ovarian cancer	NA	NA	NA	NA	NA	NA	NA	NA	NA
Lung cancer	− 0.006	0.015	0.676	− 0.002	0.014	0.877	− 0.001	0.019	0.966
Breast cancer	0.003	0.016	0.825	− 0.006	0.016	0.698	0.010	0.018	0.586
Thyroid cancer	0.010	0.035	0.779	0.037	0.037	0.321	− 0.004	0.044	0.932
Brain cancer	− 0.011	0.044	0.808	− 0.017	0.041	0.682	− 0.010	0.056	0.858

*CD* Crohn’s disease, *IBD* inflammatory bowel disease, *LDSC* linkage disequilibrium score regression, *NA* not available, *UC* ulcerative colitis

### The causal association between IBD and cancers according to Mendelian randomization analysis

In the East Asian population, the number of IVs for all considered traits ranged from 28 to 56, the *R*^2^ varied from 55.06 to 122.39%, the *F*-statistics varied from 1860 to 4217, and the statistical power varied from 6 to 53% (Additional file 1: Table S4). In the European population, the IVs ranged from 44 to 116, the *R*^2^ varied from 41.04 to 85.37%, the *F*-statistics for all traits under consideration varied between 3226 and 8196, and the statistical power varied from 5 to 91% (Additional file 1: Table S5). All *F*-statistics were greater than 10, suggesting no potential weak instrument bias.

**Fig. 3:**
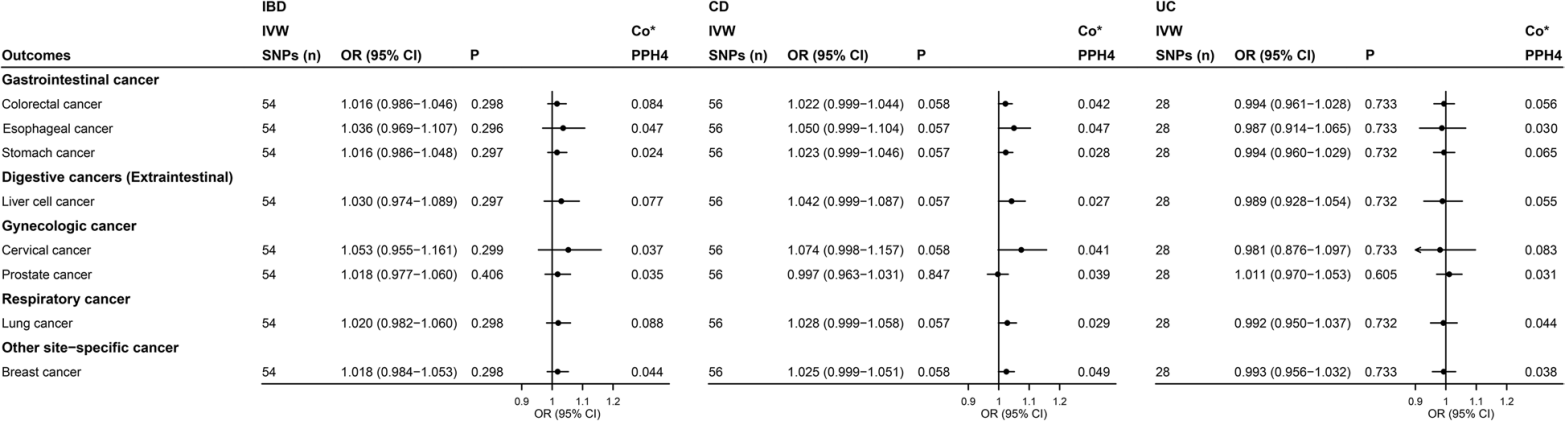
Forest plot of the results of IVW analysis and colocalization analysis for the association of IBD with cancers in the East Asian population. Co*, colocalization analysis; the average value of PPH_4_ across all regions was taken as the final colocalization result. CD, Crohn’s disease; CI, confidence interval; IBD, inflammatory bowel disease; IVW, inverse-variance-weighted; OR, odds ratio; SNPs, single nucleotide polymorphisms; UC, ulcerative colitis

According to our primary analysis of IVW data, IBD, including CD and UC, had no significant associations with any cancer in the East Asian population (all *P* values > 0.05; Fig. 3). There were some suggestive associations between CD and CRC (odds ratio [OR] = 1.036, 95% confidence interval [CI] = 1.000–1.074, *P* = 0.048) according to the WM method; between IBD and stomach cancer (OR = 1.050, 95% CI = 1.001–1.101, *P* = 0.044), CD and liver cell cancer (OR = 1.071, 95% CI = 1.001–1.145, *P* = 0.048), and between CD and lung cancer (OR = 1.048, 95% CI = 1.000–1.097, *P* = 0.049; Additional file 1: Table S6) according to the PWM method. The MR-Egger intercept and MR-PRESSO test did not reveal evidence of horizontal pleiotropy (all *P* values > 0.05; Additional file 1: Table S6). Steiger directionality test results indicated that all causal directions were correct (Additional file 1: Table S6). There were pleiotropic instrumental variables for IBD and CD, but no pleiotropic instrumental variables were identified for UC (Additional file 1: Table S7). After removing pleiotropic instrumental variables, no significant association between IBD and cancer was found using any of the MR methods (all *P* values > 0.05; Additional file 1: Table S8).

**Fig. 4:**
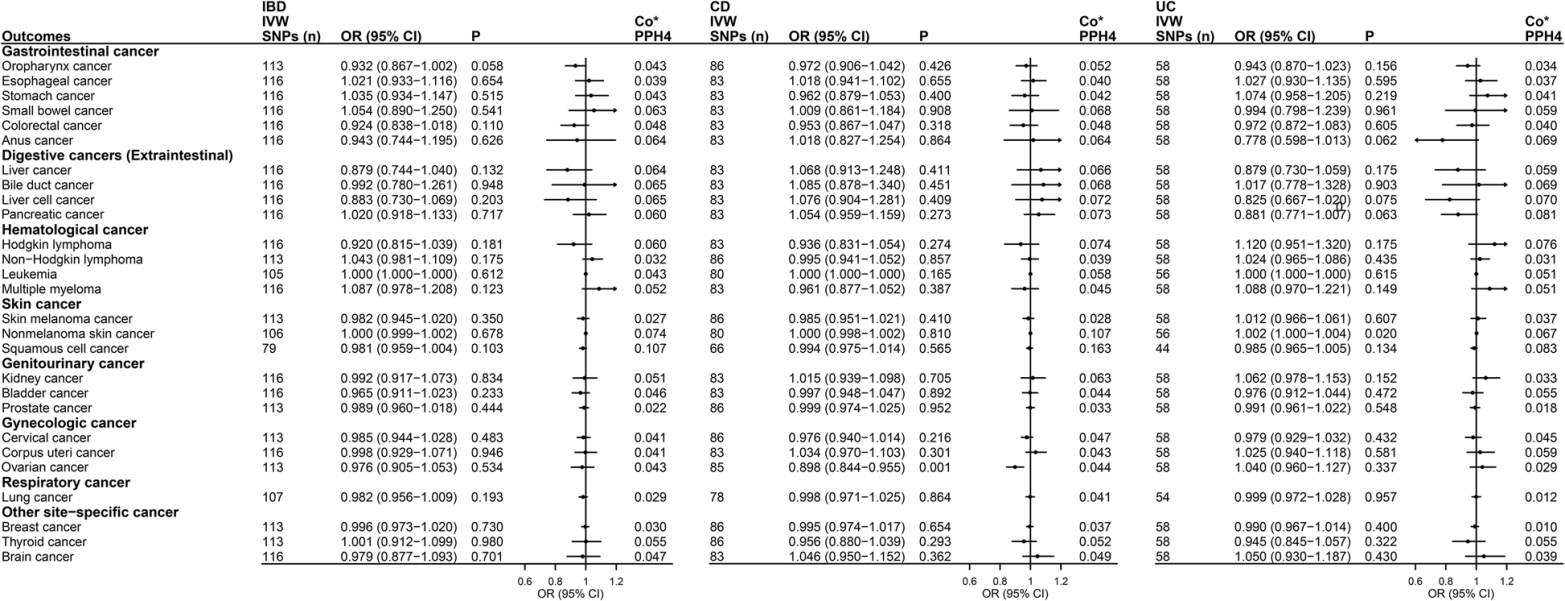
Forest plot of results from IVW analysis for the association of IBD with cancers in the European population. Co*, colocalization analysis; the average value of PPH_4_ across all regions was taken as the final colocalization result. CD, Crohn’s disease; CI, confidence interval; IBD, inflammatory bowel disease; IVW, inverse variance weighted; OR, odds ratio; SNP, single nucleotide polymorphism; UC, ulcerative colitis

The primary analysis of the IVW data revealed significant associations between CD and ovarian cancer (OR = 0.898, 95% CI = 0.844–0.955, *P* = 0.0007) and between UC and nonmelanoma skin cancer (OR = 1.002, 95% CI = 1.000–1.004, *P* = 0.019; Fig. 4) in the European population. These findings were also found in several sensitivity analyses (Additional file 1: Table S9). The MR-Egger intercept test did not reveal evidence of horizontal pleiotropy between CD and ovarian cancer (*P* = 0.739) or between UC and nonmelanoma skin cancer (*P* = 0.115). The global test *P* values from the MR-PRESSO analysis did not indicate potential pleiotropy between CD and ovarian cancer (*P* = 0.812); however, it revealed evidence of potential pleiotropy between UC and nonmelanoma skin cancer (*P* < 0.001). After removing outliers, the MR-PRESSO results still suggested a significant association between UC and nonmelanoma skin cancer (OR = 1.001, 95% CI = 1.000–1.003, *P* = 0.037). Additionally, the global test *P* values of the MR-PRESSO analysis for IBD and squamous cell cancer, as well as for UC and squamous cell cancer, were less than 0.001. After outlier exclusion, the MR-PRESSO results revealed a suggestive association between IBD and squamous cell cancer (OR = 0.970, 95% CI = 0.955–0.984, *P* = 0.0002) and between UC and squamous cell cancer (OR = 0.972, 95% CI = 0.959–0.986, *P* = 0.0003; Additional file 1: Table S9). The results of the Steiger directionality test indicated that all causal directions were correct (Additional file 1: Table S9). After removing pleiotropic instrumental variables (Additional file 1: Table S10), the MR results revealed a significant association between CD and ovarian cancer (OR = 0.890, 95% CI = 0.835–0.949, *P* = 0.0003), whereas there was no significant association between UC and nonmelanoma skin cancer (OR = 1.001, 95% CI = 0.999–1.002, *P* = 0.424; Additional file 1: Table S11). The MVMR analysis was further performed to show a lack of significant association between CD and ovarian cancer (OR = 0.940, 95% CI = 0.856–1.033, *P* = 0.201) or between UC and nonmelanoma skin cancer (OR = 0.983, 95% CI = 0.903–1.070, *P* = 0.693; Additional file 1: Table S12–13) after adjusting for confounding factors.

### Colocalization analysis for IBD and cancers

There was no shared causal variant to prove the associations of IBD, CD, or UC with cancers in East Asian or European populations using colocalization analysis based on the average value of PPH_4_ across all regions (PPH_4_ < 75%; Figs. 3 and 4). In addition, the values of PPH_4_ across each region are presented in Additional file 1: Tables S14–15. The colocalization results suggested that there may be no common biological mechanism between IBD and cancer.

### Evaluation of evidence from the MR approach

Overall, the potential biases were rated high due to pleiotropy bias, inconsistency with the sensitivity analyses, and lack of repeatability (Additional file 1: Table S3). However, we performed analyses to avoid potential pleiotropy with the use of PhenoScanner tools and the MVMR approach. In addition, we comprehensively considered the results of genetic correlation and MR and colocalization analyses and different populations.

## Discussion

After fully balancing the genetic evidence from genetic correlation, MR and colocalization analyses, we used the latest and largest datasets to investigate the causal associations of genetically determined IBD with the risk of cancers across East Asian and European populations. In the East Asian population, neither of the three approaches provided genetic evidence for the associations of IBD with eight common cancers. In the European population, we found evidence for associations between CD and ovarian cancer and between UC and nonmelanoma skin cancer via the MR method. However, these findings were not supported by the results of LDSC and colocalization analyses. Genetic backgrounds vary between different ethnicities, which might lead to differences in the results of MR analysis. However, we did not provide robust genetic evidence showing the causal associations between IBD and cancer risk in either East Asian or European populations.

Our findings were somewhat inconsistent with previous MR study results. Previous MR studies have focused on examining the association of genetically predicted IBD with intestinal cancers, including CRC, oral cancer, and pharyngeal cancer; hepatobiliary pancreatic cancer [16, 18–20]; and only one of the extraintestinal cancers, prostate cancer [17]. In addition, a recent comprehensive MR analysis exploring the associations between IBD and 32 cancers in the European population showed causal associations of predicted IBD with the oral cavity and breast cancer [42]. The different findings in these studies may be due to the use of different populations and different summary-level GWAS data for exposure and outcome. Compared to other studies, we utilized the latest and largest GWAS summary data across East Asian and European populations. In addition, we combined the genetic evidence from different methods.

To the best of our knowledge, no previous studies have systematically and comprehensively investigated the genetic association between IBD and various cancer sites, balancing genetic evidence from genetic correlation, MR, and colocalization analyses. Our study presented one result and explanation: MR analysis was significant, whereas LDSC and colocalization analyses were not, which suggested that the findings in MR may be prone to false positive bias. When the proportion of heritability explained by genome-wide significant SNPs, as determined by MR approaches, is low, the accuracy of MR results may be inferior to LDSC [30]. MR analysis is more liberal than colocalization analysis, and significant MR findings without evidence of colocalization may suggest distinct causal variants for exposure and outcome that are in linkage disequilibrium, indicating that the assumption of MR may have been violated and that the results are unreliable [29]. In addition, we evaluated and corrected pleiotropy using several methods, including MR-Egger analysis, the MR-PRESSO global test, the removal of instrumental variables associated with confounding factors, and MVMR analysis, to comprehensively assess the MR results. Overall, our findings suggest that the associations between CD and ovarian cancer and between UC and nonmelanoma skin cancer according to MR analysis may be false. Our study summarized ten possible results and explained them, filling the gap in this area by combining the evidence from genetic correlation, MR, and colocalization analyses.

Considering the pathogenesis of chronic systemic inflammation and immune dysregulation, the disorders associated with IBD extend beyond intestinal diseases, leading to an increased risk of extraintestinal manifestations in IBD patients [43]. Our results are mostly contrary to the hypothesis of pathogenesis and the findings of umbrella reviews based on observational studies [15]. The previously reported observational associations between IBD and cancer risk might be affected by detection bias, unobserved confounding factors, and pleiotropy. First, the excess diagnosis due to endoscopic screening and surveillance in patients with IBD might contribute to the rising risk of common cancers in observational studies. A Scandinavian register-based cohort study from 1969 to 2017 recently showed that the incidence of CRC increased during the first year after IBD diagnosis but significantly decreased after the first year of follow-up [44]. A greater extent of medical examinations, particularly a higher frequency of colonoscopy/sigmoidoscopy, in IBD patients than in the control population induces detection bias [45] and results in a false association between IBD and CRC. During long-term follow-up after IBD diagnosis, it is possible that the decrease in CRC incidence could be related to changes in lifestyle or medical management during the treatment of IBD. In addition, treatment following the diagnosis of IBD might contribute to the increased risk of common cancers in observational studies. A systematic review summarized the protective and risk factors for CRC in IBD patients and showed that primary sclerosing cholangitis, postinflammatory polyps, and colon segment resection were risk factors for the incidence of CRC in IBD patients [46]. Because genetically predicted IBD was also associated with a higher probability of taking anti-IBD medication, it could be that exposure to such drugs, rather than having IBD, was associated with the risk of cancer. Increasing evidence has shown that common treatments for IBD patients, such as glucocorticoids, thiopurines, and immunomodulatory agents, promote the development of cancer [47–51]. Moreover, our MR findings suggest an association between CD and a reduced risk of ovarian cancer, which could be attributed to pleiotropy. Our MVMR analysis showed an insignificant causal association between CD and ovarian cancer.

Overall, our findings suggest that IBD might not be a trigger for developing cancers, and the observed association between IBD and cancer needs to be interpreted very cautiously in clinical practice. Future studies are warranted to investigate the common risk factors or confounding factors underlying the association between IBD and cancer risk. In addition, the increased risk of cancer in observational studies might be attributed to factors accompanying the diagnosis of IBD, especially treatments for IBD patients. A full understanding of cancer as an adverse consequence of IBD is necessary to ensure appropriate vigilance against cancer. These findings should be confirmed by better-designed epidemiologic studies.

### Strengths and limitations

Our study has notable strengths. The genetic correlation, MR, and colocalization analyses have specific strengths and limitations that can complement each other to some extent to mitigate some false negative and false positive results. Our work is a landmark study in that it provides guidance on how to integrate evidence from genetics-driven studies accumulated to date to enable a more reliable interpretation of the epidemiological relationship between IBD and cancer. In addition, we used data from the latest available GWAS data with the largest sample size or the largest sample size of patients for exposure and outcome under investigation, and we repeated our findings using different methods and different ancestries. The objective estimates of the association between IBD and cancer may be more precise than previous estimates and are less prone to bias.

We acknowledge the limitations of this study. First, because either one or both traits had too low heritability, some results from LDSC analysis were not available. Second, a key assumption of MR is that SNPs are not associated with any confounders of the exposure or the outcome. Even if we considered the pleiotropic bias, any MR study could not completely rule out pleiotropic bias. Third, we investigated three exposed phenotypes and used different sets of IVs, but the power of our analyses with the different instruments varied, which could have induced weak instrumental variable bias. Furthermore, the colocalization analysis of genes and proteins is widely used but is still in the development stage for determining the optimal dichotomy. We included all IVs in the colocalization analysis and chose a cut-off value of 0.75 for PPH_4_. Besides, we used multiple data sources to increase the statistical power, but the relatively small number of cancer patients reduced the power of the MR analyses. In addition, despite leveraging data from genetic studies with very large sample sizes, our study was insufficient to detect very small effects. Moreover, we could not further adjust for potential confounders due to a lack of individual-level data. Finally, due to the limited cancer GWAS data on Asians, the results for more than half of the cancers could not be validated in the Asian population. We should carefully utilize our findings in racially and ethnically diverse populations.

## Conclusions

In summary, according to comprehensive genetic correlation, MR, and colocalization analyses, inherited lifetime exposure to IBD was not strongly associated with the risk of cancer. Exposure to IBD might not independently contribute to the risk of cancers, underlying that the increased risk of cancers observed in observational studies might be attributed to factors accompanying the diagnosis of IBD, especially treatments for IBD patients.

### Supplementary Information

**Additional file 1: Table S1.** STROBE Statement-Checklist of items in reports of Mendelian randomization. **Table S2.** Characteristics of the summary GWAS data. **Table S3.** Assessment criteria for risk of bias assessment via the MR approach. **Table S4.** Characteristics of the instrumental variables in the East Asian population. **Table S5.** Characteristics of the instrumental variables in the European population. **Table S6.** MR estimates of the causal associations between IBD and cancers in the East Asian population. **Table S7.** Related traits of genetic variants used for Mendelian randomization analysis in the East Asian population. **Table S8.** MR estimates of the causal associations between IBD and cancers after removing instrumental variables associated with confounding factors in the East Asian population. **Table S9.** MR estimates of the causal associations between IBD and cancers in the European population. **Table S10.** Related traits of genetic variants used for Mendelian randomization analysis in the European population. **Table S11.** MR estimates of the causal associations between IBD and cancers after removing instrumental variables associated with confounding factors in the European population. **Table S12.** MVMR estimates of the causal associations between CD and ovarian cancer after adjusting for confounding factors. **Table S13.** MVMR estimates of the causal associations between UC and nonmelanoma skin cancer after adjusting for confounding factors. **Table S14.** Colocalization analysis for the associations between IBD and cancers in the East Asian population. **Table S15.** Colocalization analysis for the associations between IBD and cancers in the European population.

**Additional file 2: Fig. S1.** Mendelian randomization model. Solid arrows = causal effects; dashed arrows = causal effects prohibited by MR assumptions II and III. Assumption I: Genetic instruments are associated with exposures; Assumption II: Genetic instruments are independent of confounding factors; Assumption III: Genetic instruments affect outcomes only through exposures. IVW: inverse-variance-weighted.

## Data Availability

The data underlying this article are available in the article and in its online supplementary material.

## Abbreviations

CD: Crohn’s disease
CI: Confidence interval
CRC: Colorectal cancer
GWAS: Genome-wide association study
IBD: Inflammatory bowel disease
IVs: Instrumental variables
IVW: Inverse-variance weighted
LD: Linkage disequilibrium
LDSC: Linkage disequilibrium score regression
MR: Mendelian randomization
MVMR: Multivariable Mendelian randomization
OR: Odds ratio
PP: Posterior probability
PRESSO: Pleiotropy residual sum and outlier
PWM: Penalized weighted median
RAPS: Robust adjusted profile score
*r*_*g*_: Genetic correlation
SE: Standard error
SNPs: Single nucleotide polymorphisms
UC: Ulcerative colitis
WM: Weighted median
