# Supplementary figures and images for: Association between inflammatory bowel disease and cancer risk: evidence triangulation from genetic correlation, Mendelian randomization, and colocalization analyses across East Asian and European populations
Source: BMC Med. 2024 Mar 25;22:137. doi: 10.1186/s12916-024-03352-9 (PMC10964701; doi:10.1186/s12916-024-03352-9)

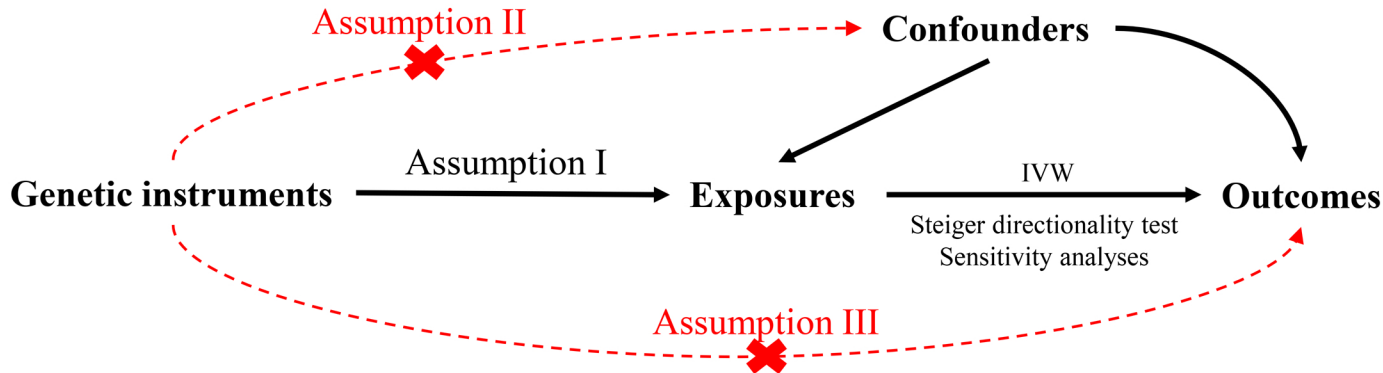

Supplement: Supplementary file 2 — Additional file 2: Fig. S1. Mendelian randomization model. Solid arrows = causal effects; dashed arrows = causal effects prohibited by MR assumptions II and III. Assumption I: Genetic instruments are associated with exposures; Assumption II: Genetic instruments are independent of confounding factors; Assumption III: Genetic instruments affect outcomes only through exposures. IVW: inverse-variance-weighted. [file 12916_2024_3352_MOESM2_ESM.pdf]
